# Measures for sustainable forest management in the tropics – A tree-ring based case study on tree growth and forest dynamics in a Central Amazonian lowland moist forest

**DOI:** 10.1371/journal.pone.0219770

**Published:** 2019-08-06

**Authors:** Martin Worbes, Jochen Schöngart

**Affiliations:** 1 Department for Crop Sciences, Georg-August-University, Grisebachstraße, Göttingen, Germany; 2 Instituto Nacional de Pesquisas da Amazônia (INPA), Manaus, Brazil; University of Oregon, UNITED STATES

## Abstract

The conservation of tropical forests is recognized as one of the most important challenges for forestry, ecology and politics. Besides strict protection, the sustainable management of natural forests should be enhanced as a key part of the foundation for the maintenance of tropical rain forest ecosystems. Due to methodological reasons it has been complicated to attain reliable growth data to plan sustainable felling cycles and rotation periods. Tree ring analyses enable the estimation of growth rates over the entire life span of trees and their age as well as giving hints from forest dynamics in previous centuries. For tree ring analysis, stem disk samples were taken from three important commercial tree species (*Cariniana micrantha*, *Caryocar villosum* and *Manilkara huber*i) in the upland (terra firme) forests of the Precious Woods Amazon logging company near Itacoatiara, Brazil. Based on radiocarbon estimates of individual growth zones, the annual nature of tree rings was proven for the three species. Tree rings were measured and the results used together with height estimates to model diameter, height and volume growth. The age of the eldest tree, a *C*. *micrantha*, was 585 yrs with 165 cm in diameter. The species’ diameter increments range from 0.20±0.12 cm yr^-1^ to 0.29±0.08 cm yr^-1^. At first sight, this is considerably lower than increments reported from other Amazonian or African timber species. Considering the respective wood density there is no significant difference in growth performance of dominant timber species across continents. The interpretation of lifetime tree ring curves indicate differences in shadow tolerance among species, the persistence of individuals in the understory for up to 150 years and natural stand dynamics without major disturbances. Management criteria should be adapted for the measured growth rates as they differed considerably from the Brazilian standards fixed by laws (felling cycle of 25–35 years and a common minimum logging diameter of 50 cm). Felling cycles should be increased to 32–51 years and minimum logging diameters to 63–123 cm depending on the species.

## Introduction

The Amazonian lowland rainforest is the largest connected rainforest in the world. It is a hotspot for biodiversity comprising some 16.000 tree species [1] among many other taxa playing a key role in the global carbon cycle and acting as a huge carbon storage base and sink [2, 3]. This unique ecosystem is endangered by an increasing demand on agricultural products in the Amazon region, an increasing population [4] and unsustainable or illegal logging [5]. In the past decade, up to 24.5 x 10^6^ m^3^ of logs per year were produced in the Brazilian Amazon region alone [6]. These activities exploit timber resources, leaving degraded secondary forests or opening the exploited areas for other land users [7, 8].

The concepts of sustainable forest management strongly contrasts this widely used practice which aims to encourage the long-term use of natural resources that promise an a way to protect tropical forest ecosystems [9].

A key measure of assurance of sustainable timber production is the knowledge of growth rates of commercial species in order to balance exploitation and regrowth [10–12]. Such data, however, are very scarce for timber species in Central Amazonian rainforests, except from a few diameter measurement studies [13–16]. Tree ring studies could balance this deficit and are, despite earlier doubts on the existence of annual rings in the tropics [17], often successfully applied in tropical ecology and forestry [18–24].

On the basis of tree-ring data the concept of Growth-Oriented Logging (GOL) for tropical timber resources in Amazonian floodplain forests was developed [12] and has been extended to other Brazilian wetlands [25–27]. The model is an approach to improve sustainable management by using species-specific and site-specific MLDs and felling cycles that replace theoretical legal regulations through realistic calculations based on empirical data.

In this study, we first prove the annual formation of tree rings for three commercial tree species (*Cariniana micrantha*, *Caryocar villosum* and *Manilkara huberi*) from the Central Amazonian lowland rainforest by radiocarbon dating. Based on tree-ring measurements we provide data on tree ages and diameter increment, we analyse long term trends of the tree ring time series to gain insights into the history of forest stands. We discuss predictors of growth rates, in particular wood density, comparing our results with those of other tropical timber species.

Through developing models for diameter, height and volume growth, we estimate species-specific MLD and felling cycles. The derived management criteria are compared with currently practised timber resource management in lowland rain forests and other ecosystems in Amazonia.

## Material and methods

### Study site

**Fig 1:**
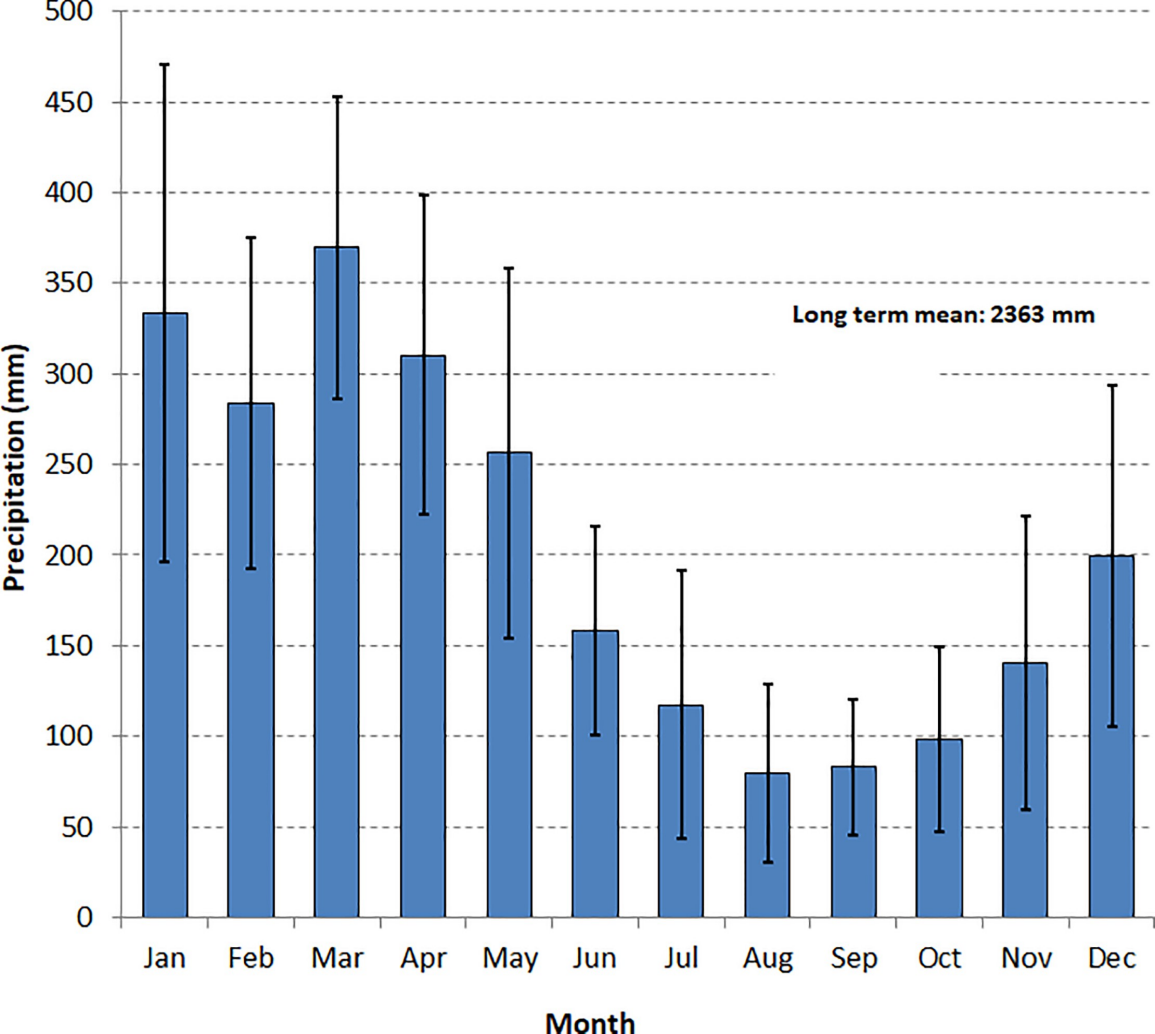
Monthly rainfall in Central Amazonia. Monthly precipitation in Itacoatiara Brazil in mm together with the respective standard deviation.

The study was performed in the managed forest of the Mil Madeiras Preciosas Ltda. Company, which is part of the Precious Woods Holding (Switzerland) located 250 km east of Manaus close to the municipality of Itacoatiara, Brazil (20^0^ 49’ S, 58^0^ 44 W’). The climate of the study area is characterized by an average temperature of about 26°C and a mean annual precipitation of 2200 mm. The distribution of the rainfall follows a strong seasonal pattern with a dry period from June to October (Fig 1). The soils are classified as yellow Latosols (Ferrasols) being nutrient-poor, with a basal saturation of about 10% and a pH-value of 3.7–4.7 [28].

An area of more than 506.000 ha of primary humid lowland (*terra firme*) forest is currently under management based on the CELOS System [29], which is characterized by reduced impact logging and other features. The forest management plan was certified in 1997 according to the FSC standards [30]. The average density of commercial species with a diameter at breast height (DBH) of more than 50 cm is about 20 trees per hectare with an average wood volume of 83.7 m^3^ ha^-1^ [31]. The number of managed commercial tree species dropped from an initially projected 80 species to less than 30 in recent years. The polycyclic management system provided a felling cycle of 30 years with a harvest volume of about 30–35 m^3^ ha^-1^. The actual harvest volume is much lower, at 10–15 m^3^ ha^-1^ [31].

### Studied tree species

The three studied species are common and frequent in humid lowland forests in Amazonia. *Caryocar villosum* (Caryocaraceae) and *Manilkara huberi* (Sapotaceae) occur throughout the Amazon region from the Guayanas in the North to Mato Grosso in the South [32]. *Cariniana micrantha*, Lecythidaceae is found in the center and the western part of the Amazon basin [33]. Itis a deciduous species with leaf exchange occurring in the late dry period from August to November and classified as shade tolerant [34]. Shade tolerance is also documented for the evergreen *M*. *huberi* [35, 36], while the opposite is mentioned for the deciduous species *C*. *villosum* [37]. All three species are described as being an element of the main canopy having a height in the studied forest of up to 40–50 m. These belong to the most frequent and important timber species of Mil Madeireira with medium to high wood densities [38, 39]. From now on the study species are referred to the genus names.

### Field sampling

The samples were taken as part of three separate excursions, having all types of permission and support from the Precious Woods Amazon Company.

1. Samples for radiocarbon dating of all investigated species were taken from the log store of the company in 1998. Stem discs were cut from the base of the trees with the largest diameters.
2. *Cariniana* samples were collected in the logging area from the base of freshly felled trees in the Compartment A1-a; Bloc 316/9692, Plot D04 and D05 from the 25^th^ to the 28^th^ of November 2003 (11 samples) and the Compartment A1-b, Block 312/9696, Plot A3 and A4 from the 1^st^ to the 3^rd^ of December 2003 (9 samples). The DBH and the height of these trees in addition to 26 standing trees were measured. Height estimations were performed using a SUUNTO (Finland) measuring device.
3. Twenty samples each were collected from *Caryocar* and *Manilkara* from the log store in July 2004.

In addition total tree height was measured from 30 (*Caryocar*) and 88 (*Manilkara*) standing individuals, distributed over a large range of diameters with a clinometer (Blume Leiss BL 6, Zeiss, Jena).

### Tree-ring analysis

The wood samples were analyzed in the Dendroecological Laboratory of the National Institute for Amazon Research (INPA) in Manaus. The cross sections were polished with sanding machines using paper of decreasing grain size from 40 to 600. Tree rings were identified by their wood anatomical structure using a stereo-microscope. Ring widths were measured with a measuring device (LINTAB, Rinntech, Heidelberg, Germany) to the nearest 0.01 mm. The results were analyzed with a tree-ring software (TSAP-Win).

### Radiocarbon-dating

Radiocarbon measurements in wood of isolated growth zones served for two different purposes: a) for direct age estimations from the centre of stem discs and b) for the proof of annual growth periodicity in general.

- Direct ^14^C age dating of old wood must take the SUESS-Effect [40] into account. The increased burning of fossil fuels since the middle of the 19th century has reduced the radiocarbon concentration (ð ^12^C/^14^C) in the atmosphere. In consequence organic material produced in that period appears older than it actually is and moreover the calibration curve from radiocarbon to calendar ages [41] fluctuates. Therefore, one radiocarbon age from between 1640 and 1950 can be linked with up to five possible calendar ages.
- The proof of annual wood formation is based on the nuclear weapon effect [42] and used for the age dating of individual growth zones grown after 1950 [43]. Rings were identified, and classified according to the scheme developed by [44], and predated to a calendar age.

Samples for all radiocarbon dating were taken with a steel chisel, exported with permission of the IBAMA and analysed by Beta Analytic Inc. by means of an Accelerator Mass Spectrometer. Laboratory numbers were: Beta 185187, 185188, 185189, 185190, 185191, 185192, and 185193. The results of the youngest (most recent) samples were reported as pCM (percent modern) and compared with a curve of atmospheric radiocarbon since 1950 [45].

### Growth modelling

The measured increment rates on each wood sample were accumulated from pith to bark to obtain individual growth curves [46] in order to describe the relationship between tree age and diameter (*dbh*) of a species fitted to a sigmoidal function [12]:

$$dbh=\frac{a}{\left(1+({\frac{b}{age})}^{c}\right)}$$ (1)

Height growth of a tree species was estimated by combining the age–diameter relationship as well as the relationship between diameter and tree height (*h*) measured in the field fitted to a non–linear regression model [46, 47].

$$h=a\times (1-\frac{1}{\left(1+a\times b\times dbh\right)})$$ (2)

Cumulative volume growth was calculated for each year with the basal area multiplied by the corresponding tree height and a common form factor of 0.6 [48]:

$$V_{t=\pi \times (\frac{{dbh}_{t}}{2}{)}^{2}\times h_{t}\times f}$$ (3)

Where *V*_*t*_ is the volume at age *t*; *dbh*_*t*_ is the diameter at age *t*; *h*_*t*_ is the tree height at age *t*, and *f* is the form factor.

Based on the simulated volume growth curve, current annual volume increments are derived to estimate the MLD and felling cycle [12]. The MLD is the corresponding diameter at the age of maximum volume increment. The felling cycle is calculated by the mean passage time through 10-cm diameter classes until the species-specific MLD.

### Growth changes

The individual tree ring time series were analyzed to identify abrupt growth changes [49]. Such sustained changes can be differentiated between positive releases and negative suppressions. The calculation procedure is described in detail in [24]. We followed the classification of growth patterns as in [50] with (a) no trend changes (b) one release (c) one suppression and (d) multiple releases and suppressions. The results are calculated per species in diameter classes.

## Results

### Growth zone structure

**Fig 2:**
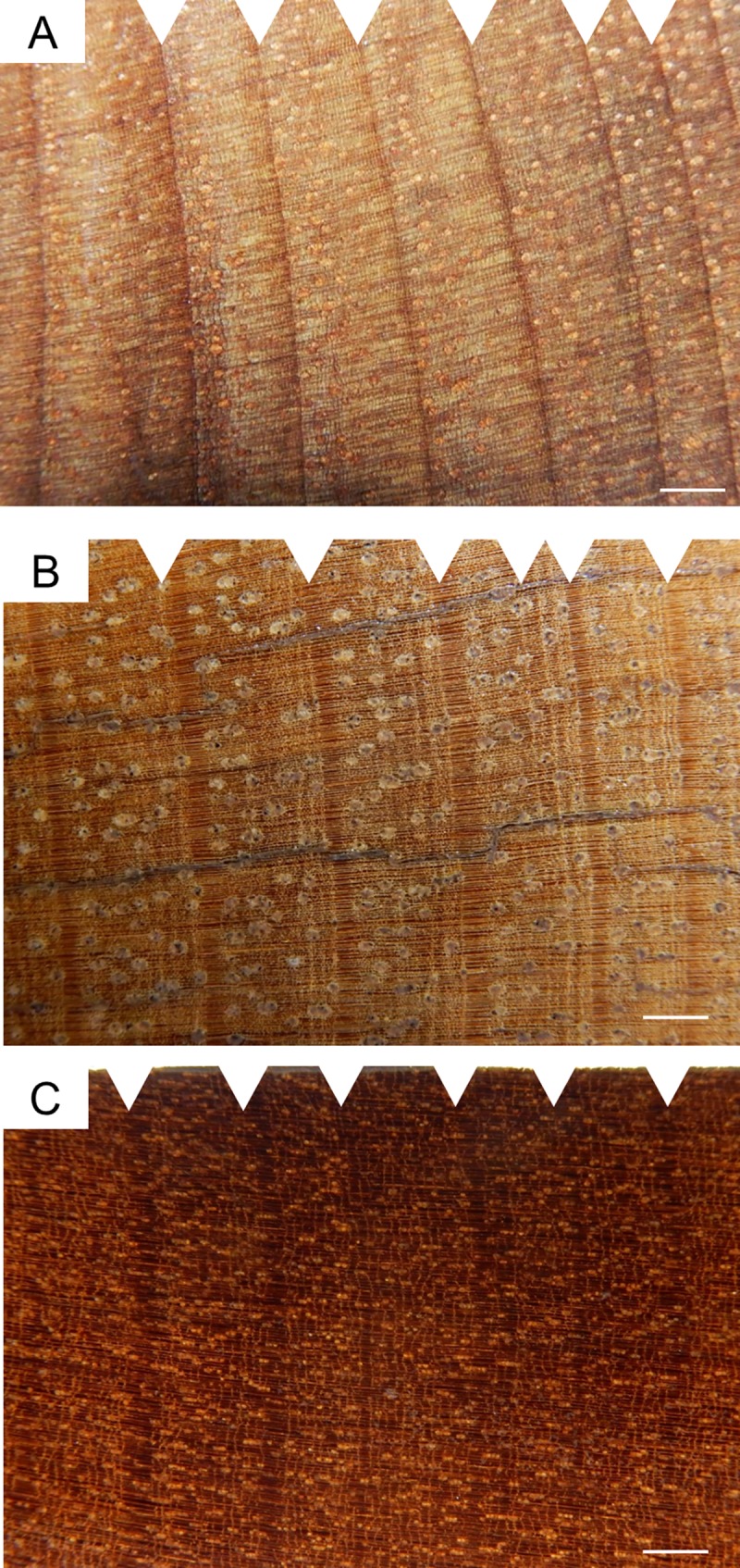
Wood cross sections of the studied species. Wood anatomical features of (A) *Cariniana micrantha* (Lecythidaceae), (B) *Caryocar villosum* (Caryocaraceae) and (C) *Manilkara huberi* (Sapotaceae) from a Central Amazonian terra firme forest. The scale is 1 mm and, the triangles mark tree ring boundaries.

The growth zone structure of *Cariniana* consists of alternating parenchyma and fiber wood bands. These bands become narrower towards the end of a growth zone, followed by a broad band of fibre tissue in the early wood. The structure of the growth zones of *Manilkara*, is almost identically with *Cariniana* with the addition of a small marginal parenchyma band between two growth zones. The growth zones of *Caryocar* are defined by an uneven vessel distribution with periodic zones of large fiber bands that are free of vessels and parenchyma (Fig 2).

### Radiocarbon dating and tree ages

The results of the radiocarbon estimates of individual growth zones in the youngest parts of the stem discs confirm the existence of annual rings in the three investigated species. The radiocarbon content of the rings from *Cariniana* matches the atmospheric curve exactly. In *Manilkara* the results of predating and radiocarbon estimation differed in the 1960s for a year. For *Caryocar*, the differences are two years (Table 1). In both cases the growth zones in that part of the disc were very small and difficult to differentiate. For all three species, the existence of annual rings opens up the possibility of age estimation through simple ring counting and to measure the tree ring width for further analysis.

**Table 1:** Radiocarbon dating.

*Species*	Tree ID Number	DBH (cm)	14C-Age (AD)	Tree ring age (AD)
*Cariniana micrantha*	45154	165	1978	1978
			1963	1963
			< 1950	1900
			**1300–1420**	**1414**
*Caryocar villosum*	47777	135	1972	1970
			1958	1960
			1948–1950	1950
			**1440–1640**	**1652**
*Manilkara huberi*	44032	91	1970	1970
			1963	1964
			1960	1960
			1950 [or 1650!!]	1950
			1930–1950	1790
			1740–1800	**1790**
			1630–1680	1790
			1530–1560	1790

Results of radiocarbon estimations of individual growth zones of three species from an Amazonian lowland forest. Values later than 1950 were compared with the atmospheric radiocarbon of [45], and before 1950 as calibrated dendro-ages [41]. The ranges given in the column “^14^C-Age” equal the 2-sigma errors of the radiocarbon measurement.

The additional radiocarbon measurements of the center of the stem discs (Table 1) partially show the influence of the SUESS effect (see methods). In *Manilkara*, we dated the sample by tree ring counting to 1790 AD. The radiocarbon estimation offers four possible age ranges at the two σ probability beginning with 1930–1950 AD until 1530–1560 AD. The range that fits the tree ring age (1740–1800) does not show a higher probability than the others. The ^14^C-result from the stem center of *Caryocar* offers a 180 year range from 1460–1640 AD, which matches the most recent period, with a tree ring age of 1652 AD. For *Cariniana* the tree ring age (1414 AD) and the range of the radiocarbon age (1300–1420 AD) match.

**Fig 3:**
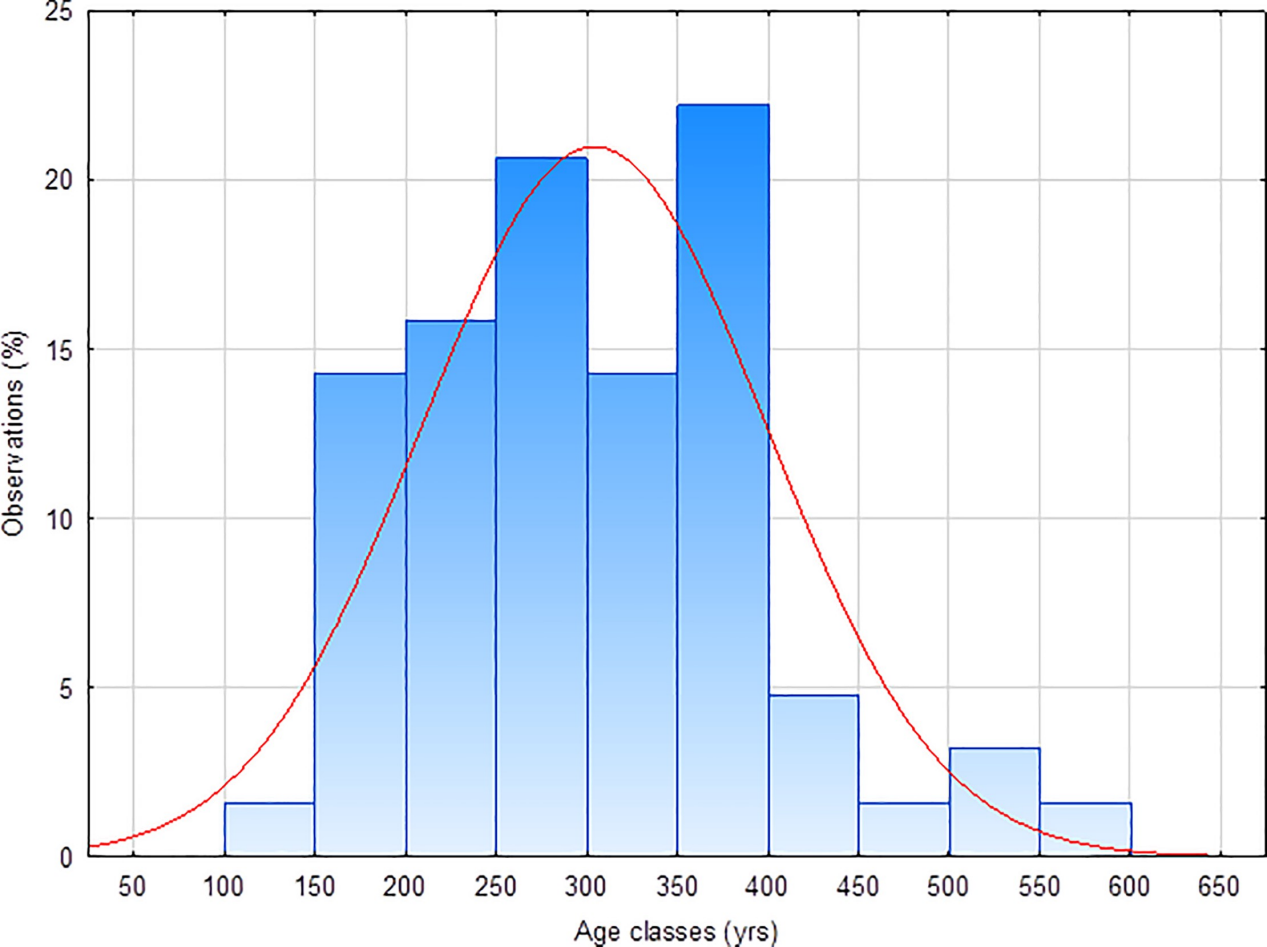
Age of all samples in classes. Age of all samples from three species of the Amazon lowland rain forest in twelve age classes. The ages are normally distributed as shown by the Gaussian curve (red line).

According to tree ring counts our oldest sample is a *Cariniana* with an age of 585 years, followed by a *Caryocar* with 546 and a *Manilkara* with 403 years (Table 2). The mean specific age varies from about 273 (*Cariniana*) to 318 years (*Caryocar*). The individual ages are randomly distributed over all species (Fig 3).

**Table 2:** Growth parameters of the studied species.

	*Cariniana micrantha*	*Caryocar villosum*	* *	*Manilkara huberi*
No. of samples	21	21		21
Mean age (yrs)	273	318		315
Max. age (yrs) and diameter of this tree (m)	585 (1.65)	546 (1.21)		403 (0.80)
Mean diameter (cm)	78.8	91.7		65.6
SD diameter	34.7	26.4		10.6
Diameter range (cm)	39–165	57–135		46–91
Mean diameter increment entire life (cm*yr^-1^)	0.29	0.27		0.20
DI (cm*yr^-1^)	0.21	0.17		0.14
Max. DI (cm*yr^-1^)	0.48	0.44		0.29
SD DI	0.08	0.07		0.04
Mean DI last 20 yrs (cm*yr-1)	0.28	0.18		0.12
Deviation from entire life DI in %	-3.4	-32.7		-40.6
Max. positive deviation %	50	10.3		5.8
Max. negative deviation %	51.4	61.3		57.6
Wood density (g*cm^-3^) [39, 51]	0.64–0.70	0.72–0.83		0.93–1.04
Increment other sources	0.27, 0.24	0.18		0.20, 0.27

Number of samples, diameter, age and diameter increment rates (DI) of three timber species from the Amazon lowland forest, Mil Madeireira, Brazil. (ns) = not significant

* significant to a 95% level.

### Growth rates

The highest specific growth rate was measured for *Cariniana* (0.29±0.08 cm yr^-1^), the lowest for *Manilkara*, (0.20±0.04 cm yr^-1^). The values for *Caryocar* are in between (0.27±0.07 cm yr^-1^, Table 2, for comparison data from other sources: [14,52,53]).

For *Cariniana* we compared the growth rates with the variables age, height and diameter of the sample trees. While the mean growth rates over the entire tree’s life correlate significantly with the height and the diameter of the trees they correlate only weak with age. The mean increments of the last 20 years do not correlate well with these parameters (S1 Table).

The specific increment is negatively correlated with wood density. For example, the slow growing *Manilkara* has the hardest wood with a density of about 1 g cm^-3^ (Table 2).

**Fig 4:**
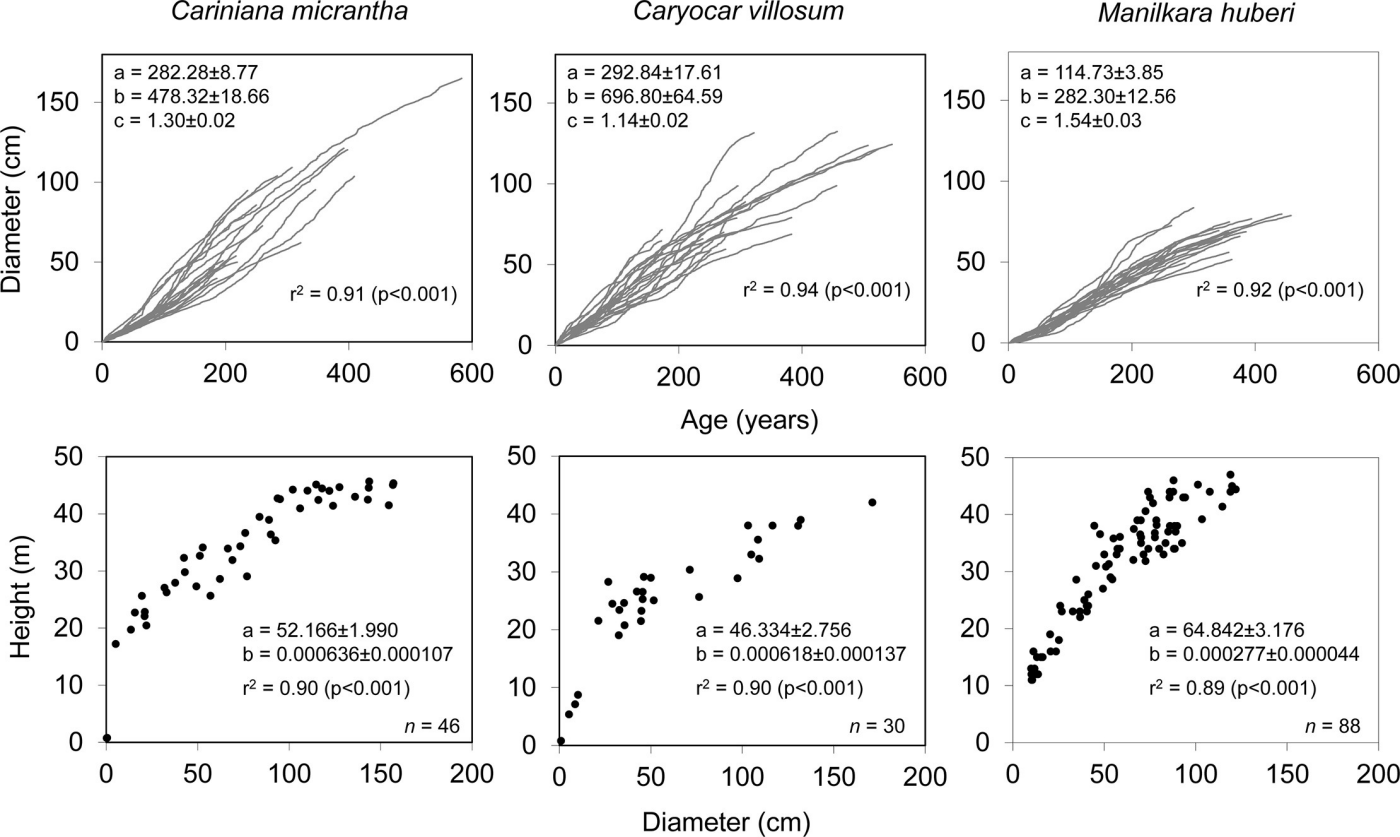
Growth functions. Non-linear relationships between age and diameter (upper panels) and diameter at breast height and tree height (lower panels) for the tree species *Cariniana micrantha* (Lecythidaceae), *Caryocar villosum* (Caryocaraceae) and *Manilkara huberi* (Sapotaceae) from Central Amazonian lowland moist forests. Correlation functions and equations in the respective graph.

The cumulative growth curves of *Cariniana* show that the best performing individual reaches the minimum logging diameter of 50 cm within 110 years, while the “slowest” needs 245 years, with the mean value at 165 years. In *Manilkara*, this spans from 163 over the mean of 212 to 307 years (Fig 4).

### Abrupt growth changes and long-term growth trends

**Fig 5:**
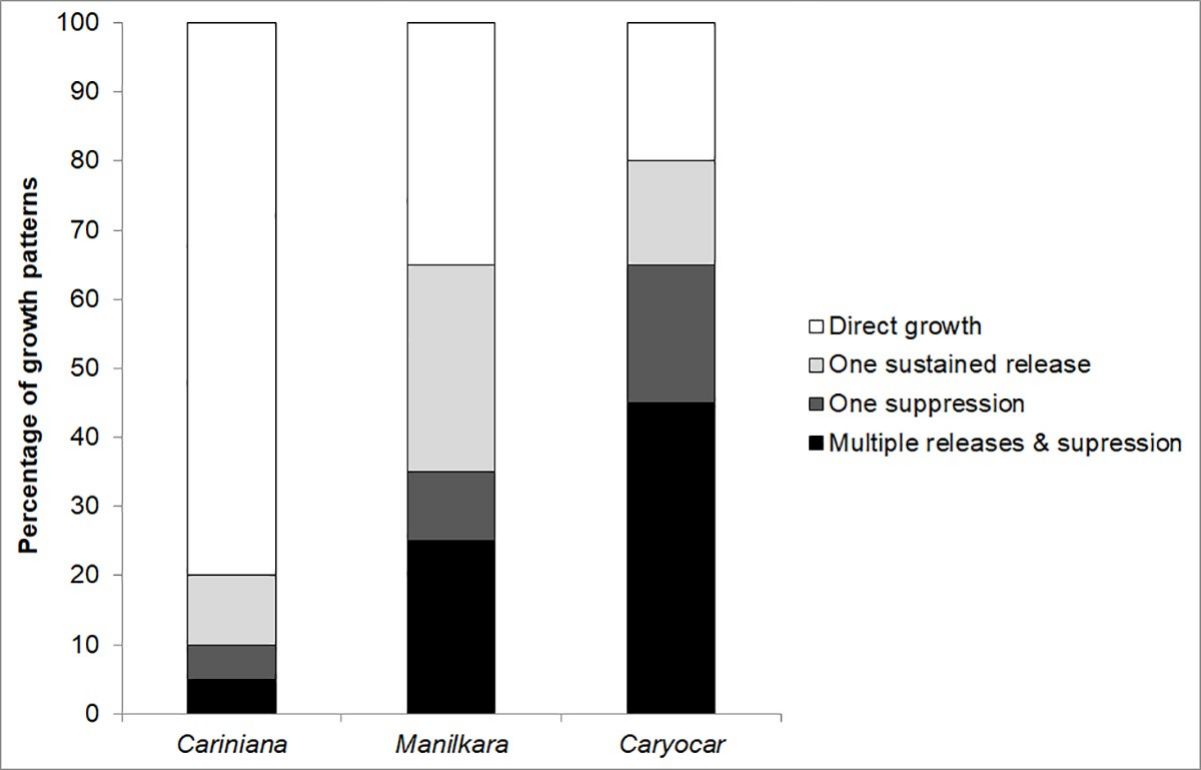
Total growth releases and suppressions. Percentage of trees from *Cariniana micrantha* (Lecythidaceae), *Manilkara huberi* (Sapotaceae) and *Caryocar villosum* (Caryocaraceae) from the Central Amazonian lowland rain forest showing either no events of suppression and release (direct growth), one suppression and release, or multiple releases and suppressions.

The mathematical interpretation of individual growth curves of *Caryocar* shows trend changes for 80% of the trees and 45% show multiple suppressions and releases. In contrast, for *Cariniana* only 20% of trees show abrupt and sustained growth trend changes (Fig 5). The growth behavior of *Manilkara* is in between these two extremes.

**Fig 6:**
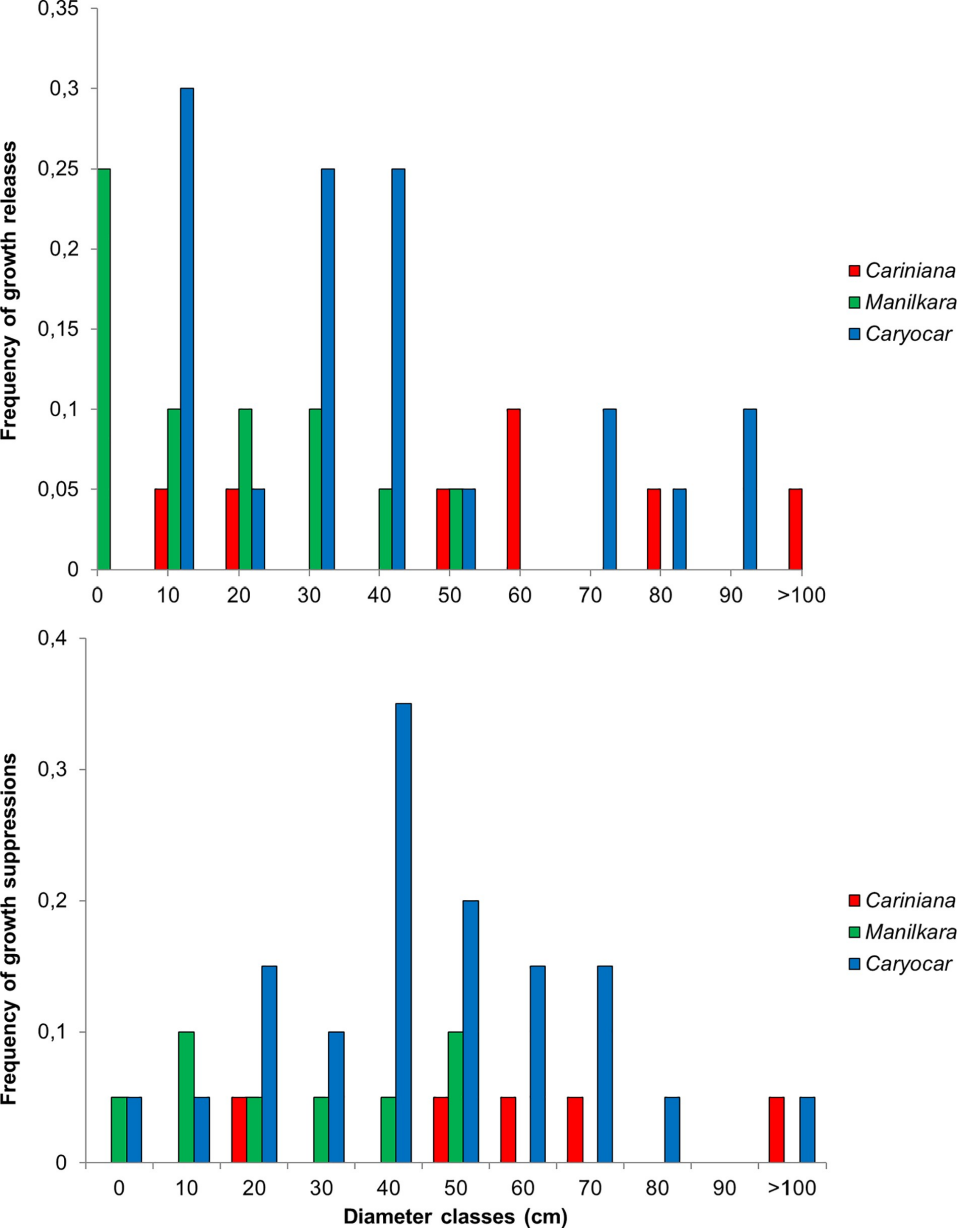
Frequency of growth releases and suppressions. Frequency of growth releases (top) and suppressions (below) in diameter classes of three tree species from the Amazonian lowland rainforest, derived from tree ring time series.

In *Cariniana*, growth suppressions and releases are randomly distributed at low frequencies over diameter classes (Fig 6). *Manilkara* presents a high frequency of growth releases in the lowest diameter classes and a decreasing frequency of such events with increasing diameters. Growth suppressions in this species are randomly distributed. In *Caryocar*, the frequency of releases is highest in the diameter classes from 20 to 50 cm, while suppressions are observed at higher frequencies at diameters from 50 to 80 cm.

**Fig 7:**
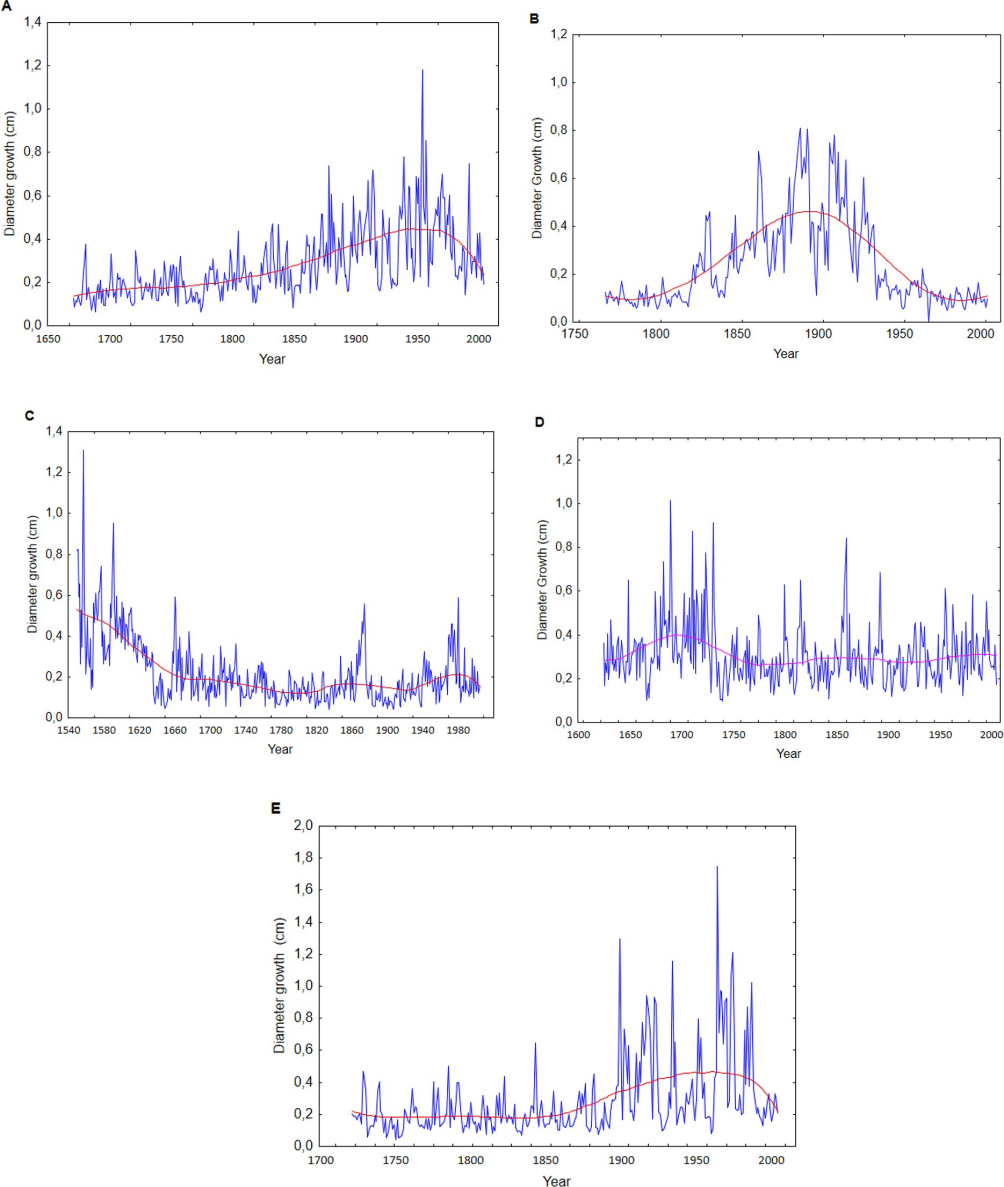
Types of growth trends. Characteristic individual trend curves. A) Understory low growth rates during the initial growth periods in the given case of about 150 years, followed by a sharp release to a much higher level (e.g. *Cariniana micrantha)*. B) Similar behavior as in A with a shorter understory period and a pronounced decrease after the release (e.g. *Manilkara huberi*). C) High growth rates in the sapling stage and continuous decline in the following stages (e.g. *Caryocar villosum*). D) Relatively stable growth levels throughout the entire lifespan (e.g. *C*. *micrantha* 4. E) Starting at a low level, followed by a constant increase without major abrupt growth changes (e.g. *C*. *micrantha*).

For the interpretation of the long term growth behavior we classified the original curves into five trend types (Fig 7). In type A, the species consistently show low increments at different, specific levels at the beginning of their life. In extreme cases, this suppression lasts up to 150 years, followed by a sharp and long lasting release (Cm 25%, Cv 30%, Mh 30%). In type B, the release is followed by a reduction of the growth rates compared to the previous level, until the youngest ring in 2004. This was only observed in *Manilkara* in 40% of individuals. In type C, early stage growth rates are very high, which later decline(Cm 5%, Cv 25%, Mh 5%). Type D, with constant growth or moderate multiple waves over the entire lifespan, is common in all species (Cm 35%, Cv 45%, Mh 25%). In type E, after a moderate start, growth rates increase constantly without major or abrupt changes (Cm 35%, Cc 0% Mh 5%).

### Growth models

#### Diameter and height growth modeling

**Fig 8:**
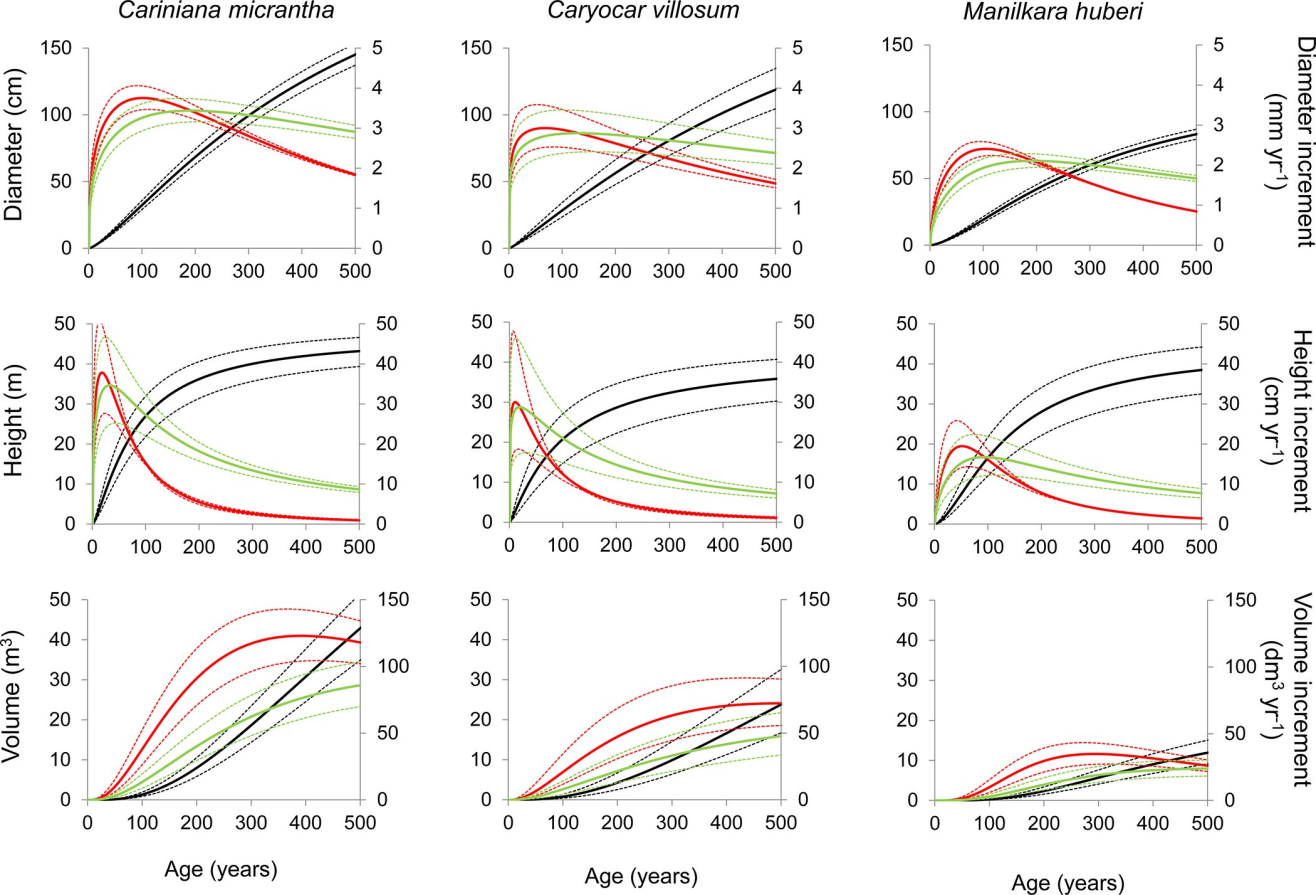
Specific growth models. Growth models in diameter (upper panels), tree height (middle panels) and volume (lower panels) for the tree species *Cariniana micrantha* (Lecythidaceae), *Caryocar villosum* (Caryocaraceae) and *Manilkara huberi* (Sapotaceae) from the Central Amazonian lowland rainforest. The black line indicates the cumulative growth in diameter, tree height and volume, the red line indicates the current increments in diameter, tree height and volume and the green lines indicate the mean increments in diameter, tree height and volume. The dotted lines indicate the standard errors.

The age-diameter relationships of *Cariniana*, *Caryocar* and *Manilkara* indicate significant correlations, 91–94% of the variation in diameter are explained by the estimated age (Fig 5). The same holds for the diameter-height relation (Fig 5). Based on mean age-size relationships we modeled cumulative diameter growth and in order to derive the current and the mean annual diameter increment (Fig 8). *Cariniana* attained the maximum current diameter increment (3.8±0.3 mm) at an age of about 101±11 years, *Carycoar* at an age of 83±17 years (3.2±0.6 mm) and *Manilkara* at an age of 88±11 years (2.5±0.3 mm). The MLD of 50 cm (Brazilian forest legislation) is achieved within 147±13 years by *Cariniana* and 170±35 years by *Carycoar*, while it took over 240±29 years for *Manilkara* to pass this limit. All species achieved maximum current height increments earlier as maximum diameter increments, at ages varying between 10±5 years (*Caryocar*) and 49±10 years (*Manilkara*) (Fig 8).

#### Volume growth, minimum logging diameters and felling cycles

While maximum current increments in height and diameter were achieved within 50 and 100 years, respectively, it took 300–400 years to reach maximum current volume increments (Fig 8). These varied considerably between 35±9 dm^3^ year^-1^ (*Manilkara*), 84±22 dm^3^ year^-1^ (*Carycoar*) and 123±20 dm^3^ year^-1^ (*Cariniana*). The corresponding age at the maximum current volume increment was defined as species-specific MLD by the age-diameter relationship. All species presented higher MLDs compared to the legal norms (50 cm), differing between 63±6 cm for *Manilkara*, 101±13 cm for *Caryocar* and 123±6 cm for *Cariniana* (Table 3). The estimated felling cycles of the mean passage time through 10-cm diameter classes until reaching the defined MLDs were 32±2 years (*Cariniana)* 39±5 years (*Caryocar*) and 51±5 years (*Manilkara*).

**Table 3:** Wood density and management criteria for timber species in the Amazon region.

Tree species	Wood density (g cm^-3^)^1^	Age at 50 cm MLD (years)	Mean DG at 50 cm diameter (cm yr^-1^)	MLD (cm)	Felling cycle (years)
** **	**Central Amazonian lowland forest**^2^				
*Cariniana micrantha*	0.55–0.64	147	0.34	123	32
*Caryocar villosum*	0.80–0.85	170	0.29	101	39
*Manilkara huberi*	0.90–1.00	240	0.21	63	51
*Bertholletia excelsa*	0,64	107–121	0.44	-	-
** **	**Southern and Southwestern Amazonian lowland forests**^3^				
*Amburana cearensis*	0.52	95	0.53	-	-
*Cedrela odorata*	0.47	50	1.00	-	-
*Cedrelinga catenaeformis*	0.50	61	0.82	-	-
*Goupia glabra*	0.87	107	0.47		
*Peltogyne* cf. *heterophylla*^2^	0,87	135–150	0.35	-	-
*Schizolobium parayba* var. *amazonicum*	0.47	31	1.61	-	-
*Swietenia macrophylla*^*7*,*8*^	0.51	84	0.60	-	-
Mean	0.60	81.6	0.77		
** **	**Central Amazonian white-water floodplains**^4^				
*17 species*	0.57	75,00		38–129	3–32
** **	**Central Amazonian black-water floodplains**^5^				
3 species	0.63	216		55–83	39–53

Wood densities, age at 50-cm minimum logging diameter (MLD defined by Brazilian forest legislation), minimum logging diameter and felling cycle derived from growth models for commercial tree species from different ecosystems in the Amazon basin (Brazil and Bolivia).

^1^data obtained from the Global Wood Density Database [75], indicating the average for tropical South America and own measurements.

^2^this study and [5]

^3^: [11, 19, 20, 68]

^4^: [12, 25, 27, 77]

^5^: [25, 77]

## Discussion

### Evidence of tree ring formation in an Amazonian lowland forest

Tree ring formation in tropical trees has been neglected for long time due to an assumed lack of seasonal climate and growth patterns [17]. However annual ring formation was unequivocally proven about 100 years ago on Java by [54, 55]. This is confirmed that trees on all tropical continents in various climate types are influenced by slight variations in precipitation, which triggers annual growth patterns and induces annual ring formation [16, 24, 40].

For Central Amazonia, previous tree ring research focused on floodplain trees in which ring formation is the consequence of annual inundation [40, 44]. In non-flooded lowland forest, the major ecosystem of the Amazon region, tree ring research played only a minor role [19, 56–58]. As a consequence knowledge on growth rates of Amazonian timber species is rather poor.

Our results of the radiocarbon datings confirm the existence of annual rings for the studied species and with a high probability for species in Central Amazonian lowland forests in general, as a consequence of the seasonal rainfall pattern. This opens the possibility of easy age dating and tree ring measurement to get significant data on growth rates of timber species and history of natural forest stands, That helps better understanding Tropical forest dynamics as a precondition for sustainable and considerate forest management systems.

### Forest dynamics

The oldest tree in our sample is a *Cariniana* with an age of 585 years. We found the sample at the Mil Madeira Preciosas timber store. This disc had a diameter of 1.65 m, which is only 15 cm smaller than that of the same species and location reported to be about 1370 years old [53]). This old age is a clear outlier from other reliable results for *Cariniana* in the same study (420–720 yrs), which was unfortunately never confirmed.

Due to its relatively low growth rates and high wood density the potential for *Manilkara* is high becoming old in exceptional cases. Our sample set is comprised of individuals with a comparably small diameter of up to 0.80 m, which is equally our oldest tree of that species. Extrapolating this result to an individual of 1.4 m DBH, reported from a forest in East Amazonia [36], *Manilkara* may reach an age of about 700 years. This is close to the calculated maximum age of the extremely slow growing ironwood from Borneo [59] of 900 years.

In total we found only a few individuals in the range between 500 and 600 years old while the mean age of the main cohort is much lower [60]. This reflects the typical age distribution of a dominating and ageing population of a species in a mature tropical lowland forest. A similar finding is evaluated for a *Microberlinia bisulcata* population in an old growth Cameroonian Forest [61]. For *Swietenia macrophylla*, another Amazonian long living pioneer, the maximum age of the main cohort does not exceed 150 to 200 years [19, 62].

The interpretation of a tree ring time series gives insights into the growth behavior of individuals and species almost from germination until the time of cutting or natural death. This reflects general growth conditions and individual responses over the entirety of the tree’s life for a period of up to several hundred years. This is a clear advantage compared with classical attempts based on repeated monitoring, which can only cover a few decades.

In addition to the estimation of a tree’s age, tree ring time series can give information on annual variations of rainfall [63, 64], changes of competition and occurrence of temporal events in the past. All of this information hints towards species’ growth strategies and forest dynamics in general.

The three species studied are frequent and widespread in Amazonia. *Cariniana* and *Caryocar*, with moderate wood densities and relatively high growth rates, can be classified as long living pioneers, which are often site dominant and important timber species in lowland rain forests [9, 65]. In contrast, *Manilkara* is a typical mature forest tree, due to its high wood density, slow growth and shade tolerance [66]. The latter feature, together with higher growth rates, characterizes the growth strategy of *Cariniana*. Multiple growth suppressions and releases of the growth curves of *Caryocar* in particular in low diameter classes are expressions of a high sensitivity toward competition in a closed forest stand and support its description as shade intolerant [67].

The concentration of growth changes in smaller diameter classes is a clear expression of individual competition [68]. Many individuals of all species were influenced by gap dynamics, either when germinating in the open under good light conditions (*Caryocar*) or when profiting from tree falls in the direct vicinity after a long period of suppression in the understorey (*Cariniana* and *Manilkara*). Together with the normal distribution of trees ages, this hints towards patch dynamics, [69, 70] in contrast to the influence of catastrophic disturbances as it is discussed elsewhere for a Cameroonian lowland forest [71] or a forest stand in Thailand [72].

### Predictors of growth rates

The species studied show moderate diameter growth rates (0.20–0.29 cm yr^-1^) when compared with those of other tropical timber, such as *Swietenia macrophylla*, with 0.48 to 0.84 cm yr^-1^ [19]. For Cameroonian forests in West Africa tree ring based growth rates on timber species [22, 71] exceed our values in some cases four-fold. Tree species from the nutrient-rich Amazon flood plain forest also grow faster than our species from the non-flooded forest [58].

In total, growth rates of Central Amazonian timber species seem to be in the lowest reported range for lowland humid forests. Possible influencing factors for the observed differences are light competition, nutrient availability, water supply and finally the species-specific factor wood density.

**Fig 9:**
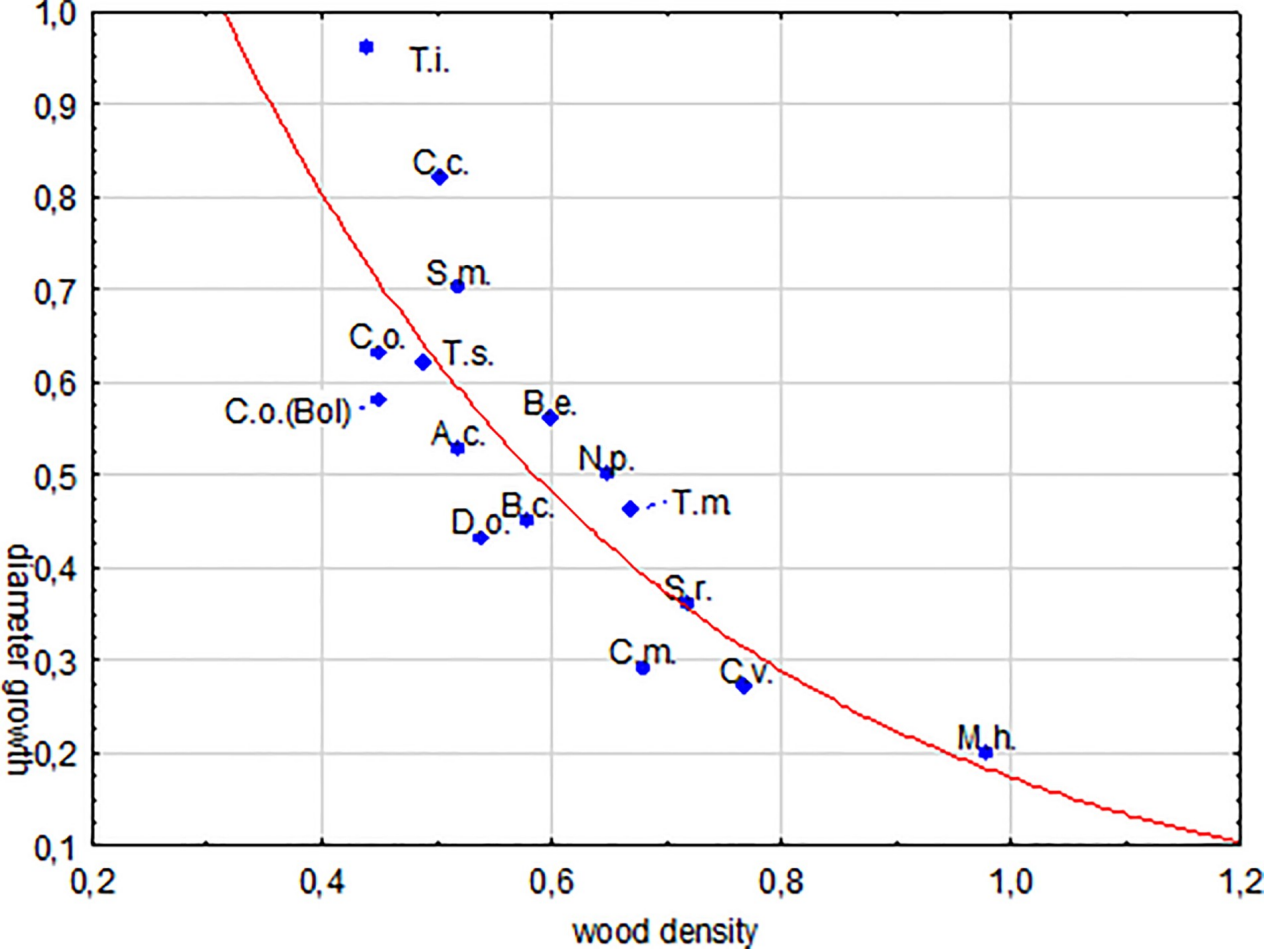
Diameter growth and wood density in African and Amazonian timber species. Correlation between diameter growth (cm*yr^-1^) and wood density (g*cm^-3^) of selected timber species from Amazonia and West Africa (r = -0.81, significance level p = 0.0001, model: growth = 2.2455*exp(-2.5652*x)). Data from [22]: *Terminalia ivorensis*, (T.i.), *Brachystegia eurycoma* (B.e.), *B*. *cynometra* (B.c.), *Daniellia ogea* (D.o.); [71]: *Triplochiton scleroxylon* (T.s.), *Sterculia rhinopetala* (S.r.) *Nesogordonia papaverifera* (N.p.), *Trilepsium madagascariense* (T.m.);) [19]: *Swietenia macrophylla* (S.m.), *Cedrela odorata* (C.o.); [11]: *Amburana cearensis* (A.c.), *Cedrela odorata* (C.o.Bol), *Cedrelinga catenaeformis* (C.c.); This study:*Cariniana micrantha* (C.m.), *Caryocar villosum* (C.v.), *Manilkara huberi* (M.h.). Growth data consists of mean values from 18 individuals or more. Wood density data: own measurements and those of the Global Wood Density Database [75].

All sites mentioned above, with the exception of the Amazonian floodplain forests belong to humid lowland forests with relatively nutrient poor soils. The samples were collected mostly in a similar way with a focus on harvested timber logs, representing dominant individuals in their respective stands. However, the species compared represent a wide range of wood densities. It is basic knowledge that pioneer trees grow fast at the expense of wood stability and, that tree species from late successional stages might behave in the opposite way. The conclusion of the general relation between both parameters is however not fully accepted [73] nor is a positive correlation assumed [74]. In contrast, we showed earlier [12] the significantly negative correlation between growth rate and wood density within and among species from Amazonian floodplain forests. Here we test this relationship for frequent species of wet lowland forests in Amazonia and West Africa. The correlation between specific means of wood density and growth rates derived from tree ring studies is highly significant (r = 0.81, Fig 9). This relationship explains the low diameter increments of our Amazonian timber species in comparison with those from other Neotropical or African sites simply through wood density differences.

### Felling cycles and minimum logging diameters

The careful and reliable estimation of specific growth rates is the basis for the modeling and calculation of felling cycles and MLDs for our Central Amazonian timber species. Felling cycles and MLDs derived from the growth models are in the range of 32–51 years and 63–123 cm, respectively, for the three species tested. This differs considerably from the criteria currently applied in the forest concessions based on Brazilian forest legislations, which condole a felling cycle of 30 years and MLD of 50 cm. This demonstrates that forest management must consider species-specific growth variations to attain a higher level of sustainability [12, 76–78] as well as differences in site conditions. For example, timber species from the nutrient-rich floodplain forests or from non-flooded forests of the southern and southwestern part of Amazonia achieve the MLD of 50 cm much faster than species. from an oligotrophic floodplain system of the Rio Negro which behave similarly to the tree species in our study (Table 3). In the same way, in all environments, the period needed to reach the MLD of 50 cm, as well as the felling cycles, tends to increase with increased wood density (Table 3). However, estimated MLDs and felling cycles can only increase the sustainability of managing timber stocks if the exploited species have the capacity to be establish after selective logging and if the remaining population is able to replace the harvested volume [77]. It is therefore of the up most importance to obtain further information on the effects of light conditions, nutrient supply and water availability on germination, establishment and growth of these tree species. Furthermore, there is an urgent need to incorporate demographic data of tree populations obtained from permanent monitoring plots) into yield projections to simulate and evaluate the impact of current management practices and to test projected MLDs and felling cycles [71, 78].

## Conclusions

The tree ring-based estimation of growth rates and analysis of tree ring time series fills gaps in understanding the specific growth behavior of tropical trees and forest stand dynamics. Modeling volume growth in time on this basis helps foresters and legislative bodies to adapt the framework of conditions for the sustainable management of related systems. For future studies, the knowledge of specific regeneration and of available resources in particular using data from forest inventories is an essential prerequisite to calculate harvest intensities and rotation periods in order to combine the management and protection of tropical rain forests.

## Supporting information

S1 Table Cross correlation of tree parameter from 20 Ca*riniana micrantha* individuals. Correlation coefficients with asterisk are significant to the p = 0.05 level.
(DOCX)

## References

[1] ter Steege H, Pitman NCA, Sabatier D, Baraloto C, Salamão RP, Gueva JE et al Hyperdominance in the Amazonian tree flora. Science. 2013;342, 1243092:1–8 10.1126/science.1243092 24136971

[2] Malhi Y. The carbon balance of tropical forest regions, 1990–2005. Current Opinion in Environmental Sustainability. 2010;2:237–44.

[3] Wagner FH, Hérault B, Bonal D, Stahl C, Anderson LO, Baker TR, et al Climate seasonality limits leaf carbon assimilation and wood productivity in tropical forests. Biogeosciences. 2016;13(8):2537–62.

[4] Fernside PM. Deforestation of the Brazilian Amazon In: Shugart H editor. Oxford Research Encyclopedia of Environmental Sciende. Oxford University Press, New York 2017 10.1093/acrefore/9780199389414.013.102

[5] Asner GP, Knapp DE, Broadbent EN, Oliveira PJC, Keller M, Silva JNM. Selective Logging in the Brazilian Amazon. Science. 2005;310:480–2. 10.1126/science.1118051 16239474

[6] SFB/IMAZON. A atividade madeireira na Amazônia brasileira: produção, receita e mercados Serviço Florestal Brasileiro (SFB), Instituto do Homem e Meio Ambiente da Amazônia (IMAZON), Belém, Brasil 2010.

[7] Putz FE, Sist P, Fredericksen T, Dykstra D. Reduced-impact logging: Challenges and opportunities. Forest Ecology and Management. 2008;256:1427–33.

[8] Edwards DP, Tobias JA, Sheil D, Meijaard E, Laurance WF. Maintaining ecosystem function and services in logged tropical forests. Trends in Ecology and Evolution. 2014;29(9):511–20. 10.1016/j.tree.2014.07.003 25092495

[9] Lamprecht H. Silviculture in the Tropics: Tropical Forest Ecosystems and their Tree Species—Possibilities and Methods for their Long-Term Utilization. GTZ, Eschborn, Germany; 1989

[10] Boot RGA, Gullison RE. Approaches to developing sustainable extraction systems for tropical forest products. Ecological Applications. 1995;5(4):896–903.

[11] Brienen RJW, Zuidema PA. The use of tree rings in tropical forest management: Projecting timber yields of four Bolivian tree species. Forest Ecology and Management. 2006B;226(1–3):256–67.

[12] Schöngart J. Growth-Oriented Logging (GOL): A new concept towards sustainable forest management in Central Amazonian várzea floodplains. Forest Ecology and Management. 2008;256:46–58.

[13] da Silva RP, Santos J, Tribuzy ES, Chambers JQ, Nakamura S, Higuchi N. Diameter increment and growth patterns for individual tree growing in Central Amazon, Brazil. Forest Ecology and Management. 2002;166:295–301.

[14] Laurance WF, Nascimento HEM, Laurance SG, Condit R, D’Angelo S, Andrade A. Inferred longevity of Amazonian rainforest trees based on a long-term demographic study. Forest Ecology and Management. 2004;190:131–43.

[15] Worbes M, Junk WJ. How old are tropical trees? The persistence of a myth. IAWA Journal. 1999;20(3):255–60.

[16] Brienen RJW, Schöngart J, Zuidema PA. Tree rings in the tropics: insights into the ecology and climate sensitivity of tropical trees In: Goldstein G, Santiago LS, editors. Tropical tree physiology: adaptations and responses in a changing environment. Springer International Publishing; 2016 p. 439–61.

[17] Whitmore TC. An Introduction to Tropical Rain Forests. Oxford: Clarendon Press; 1990.

[18] Worbes M. Annual growth rings, rainfall-dependent growth and long-term growth patterns of tropical trees from the Caparo Forest Reserve in Venezuela. Journal of Ecology. 1999;87:391–403.

[19] Dünisch O, Montóia VR, Bauch J. Dendroecological investigations on *Swietenia macrophylla* King and *Cedrela odorata* L. (Meliaceae) in the central Amazon. Trees. 2003;17(3):244–50.

[20] Free CM, Landis RM, Grogan J, Schulze MD, Lentini M, Dünisch O. Management implications of long-term tree growth and mortality rates: A modeling study of big-leaf mahogany (*Swietenia macrophylla*) in the Brazilian Amazon. Forest Ecology and Management.2014; 330: 46–54.

[21] Vlam M, Baker PJ, Bunyavejchewin S, Zuidema PA. Temperature and rainfall strongly drive temporal growth variation in Asian tropical forest trees. Oecologia. 2014;174:1449–61. 10.1007/s00442-013-2846-x 24352845

[22] Groenendijk P, Sass-Klaassen U, Bongers F, Zuidema PA. Potential of tree-ring analysis in a wet tropical forest: A case study on 22 commercial tree species in Central Africa. Forest Ecology and Management. 2014;323:65–78.

[23] Cunha TA, Finger CAG, Hasenauer H. Tree basal area increment models for *Cedrela*, *Amburana*, *Copaifera* and *Swietenia* growing in the Amazon rain forests. Forest Ecology and Management. 2016;304:174–83.

[24] Schöngart J, Bräuning A, Barbosa ACMC, Lisi SG, Oliveira JM. Dendroecological studies in the Neotropics: History, status and future challenges In: Amoroso MM, Daniels LD, Baker PJ, Camarero JJ, editors. Dendroecology. Springer International Publishing; 2017 p. 35–73.

[25] da Fonseca SF, Piedade MTF, Schöngart J. Wood growth of *Tabebuia barbata* (E. Mey.) Sandwith (Bignoniaceae) and *Vatairea guianensis* Aubl. (Fabaceae) in Central Amazonian black-water (igapó) and white-water (várzea) floodplain forests. Trees–Structure and Function. 2009;23(1):127–34.

[26] Schöngart J, Arieira J, Fortes CF, Arruda EC, Nunes da Cunha C. Age-related and stand-wise estimates of carbon stocks and sequestration in the aboveground coarse wood biomass of wetland forests in the northern Pantanal, Brazil. Biogeosciences. 2011;8:3407–21.

[27] Rosa SA, Barbosa ACMC, Junk WJ, Nunes da Cunha C, Piedade MTF, Scabin AB et al Growth models based on tree-ring data for the Neotropical tree species *Calophyllum brasiliense* across different Brazilian wetlands: implications for conservation and management. Trees. 2017;31:729–42.

[28] Quesada CA, Lloyd J, Anderson LO, Fyllas NM, Schwarz M, Czimczik CI. Soils of Amazonia with particular reference to the RAINFOR sites Biogeosciences. 2011;8:1415–40.

[29] de Graaf NR, Poels RLH. The CELOS management system: a polycyclic method for sustained timber production in South American rain forest In: Anderson AB, editor. Alternatives to deforestation: steps towards sustainable use of the Amazon rain forest. New York: Columbia University Press; 1990 p. 116–27.

[30] FSC. Principles and Criteria for Forest Stewardship (FSC-STD-01-001 V5-2 EN). https://ic.fsc.org/ 2015

[31] Precious Woods. Annual report 2017. http://www.preciouswoods.com

[32] Procópio LC, Gayot M, Sist P, Ferraz IDK. "Tauari" species (Lecythidaceae) in non-flooded Amazon forest: patterns of geographic distribution, abundance, and implications for conservation. Acta Botanica Brasilica 2010;24(4):883–97.

[33] Prance GT. The floristic compositions of the forests of Central Amazonian Brazil In: Gentry AH, editor. Four Neotropical Forests, Yale University Press 1990; 112–40.

[34] Boot R, Werger MJA, Ulloa MU. Regeneration of timber trees in a logged tropical forest in North Bolivia. Forest Ecology and Management. 2004;200: 39–48.

[35] Lorenzi H. Árvores brasileiras: manual de identificação e cultivo de plantas arbóreas do Brasil. Nova Odessa: Instituto Plantarum; 2002.

[36] Castro TC, Carvalho JOP. Dinâmica da população de Manilkara huberi (Ducke) A. Chev. durante 26 anos após a exploração florestal em uma área de terra firme na Amazônia Brasileira. Ciência Florestal. 2014;24(1):161–9.

[37] Clay JW, Clement CR (eds). 1993. Selected Species and Strategies to Enhance Income Generation from Amazonian Forests. Misc/93/6 Working Paper, Forestry Dept., FAO, Rome

[38] Loureiro AA, Silva MF. Catálogo das madeiras da Amazónia. Belém: SUDAM; 1968.

[39] Fearnside PM. Wood density for estimating forest biomass in Brazilian Amazonia. Forest Ecoloy and Management. 1997;90:59–87.

[40] Worbes M. One hundred years of tree ring research in the tropics—a brief history and an outlook to future challenges. Dendrochronologia 2002;20:217–31.

[41] Stuiver M, Reimer PJ, Bard E, Beck JW, Burr GS, Hughen KA et al INTCAL98 Radiocarbon Age Calibration, 24000–0 cal BP. Radiocarbon. 1998;40(3):1041–83.

[42] Nydal R, Lövseth K. Prospective decrease in atmospheric radiocarbon. Journal of Geophysical Research. 1970;75(12):2271–78.

[43] Worbes M, Junk WJ. Dating tropical trees by means of ^14^c from bomb tests. Ecology. 1989;70(2):503–7.

[44] Worbes M. Growth rings, increment and age of tree in inundation forest, savannas and a mountain forest in the Neotropics. IAWA Bulletin. 1989;10(2):109–22.

[45] Hua Q, Barbetti M, Rakowsky AZ. Atmospheric radiocarbon for the period 1950–2010. Radiocarbon. 2013;55:2059–72.

[46] Schöngart J, Wittmann F, Worbes M, Piedade MTF, Krambeck HJ, Junk WJ. Management criteria for Ficus insipida Willd. (Moraceae) in Amazonian white-water floodplain forests defined by tree-ring analysis. Annals of Forest Science. 2007;64:657–64.

[47] Nebel G. Minquartia guianensis Aubl.: Use, ecology and management in forestry and agroforestry. Wood density for estimating forest biomass in Brazilian Amazonia. Forest Ecology and Management. 2001;150(1–2):115–24.

[48] Cannell MGR. Woody biomass of forest stands. Forest Ecology and Management. 1984;8: 299–312.

[49] Nowacki GJ, Abrams MD. Radial growth averaging criteria for reconstructing disturbance histories from presettlement‐origin oaks. Ecological Monographs. 1997;67(2):225–49.

[50] Brienen RJW, Zuidema PA. Lifetime growth patterns and ages of Bolivian rain forest trees obtained by tree ring analysis. Journal of Ecology. 2006A;94:481–93.

[51] Loureiro AA, da Silva MF. Catálogo das Madeiras da Amazônia. I/II Belém (SUDAM): 1968:411–33

[52] Vieira S, Trumbore S, Camargo PB, Selhorst D, Chambers JQ, Higuchi N et al *Slow growth rates of Amazonian trees*: *Consequences for carbon cycling*. PNAS. 2005;102(51):18502–7. 10.1073/pnas.0505966102 16339903 PMC1310511

[53] Chambers JQ, Higuchi N, Schimmel JP. Ancient trees in Amazonia. Nature. 1998;391:135–6.

[54] Coster C. Zur Anatomie und Physiologie der Zuwachszonen und Jahresringbildung in den Tropen. Ann Jard Bot Buitenzorg. 1927;37:49–161.

[55] Coster C. Zur Anatomie und Physiologie der Zuwachszonen und Jahresringbildung in den Tropen. Ann Jard Bot Buitenzorg. 1928;38:1–114.

[56] Vetter RE, Botosso PC. Remarks on age and growth rate determination of Amazonian trees. IAWA BulIetin. 1989;10(2):133–45.

[57] Brienen RJW, Zuidema PA. Relating tree growth to rainfall in Bolivian rain forests: a test for six species using tree ring analysis. Oecologia. 2005;146:1–12. 10.1007/s00442-005-0160-y 16012820

[58] Schöngart J, Gribel R, Fonseca SF, Haugaasen T. Age and growth patterns of Brazil nut trees (*Bertholletia excelsa* Bonpl.) in Amazonia, Brazil. Biotropica. 2015;47(5):550–8.

[59] Kurokawa H, Yoshida T, Nakamura T, Nakashizuka T. The age of tropical rain-forest canopy species, Borneo ironwood (*Eusideroxylon zwageri*), determined by ^14^C dating. Journal of Tropical Ecology. 2003;19:1–7.

[60] Loehle C. Tree life history strategies: The role of defenses. Canadian Journal of Forest Research. 1988;18(2):209–22.

[61] Newbery DM, van der Burgt XM, Worbes M, Chuyong GB. Transient dominance in a central African rain forest. Ecological Monographs. 2013;83:339–82

[62] Grogan J, Landis RM, Free CM, Schulze MD, Lentini M, Ashton MS. Bigleaf mahogany *Swietenia macrophylla* population dynamics and implications for sustainable management. Journal of Applied Ecology. 2014;51(3):664–74.

[63] Berlage HP. Over het verband tusschen de dikte der jaarringen van djatiboomen (*Tectona grandis* L. f.) en den regenval op Java. Tectona. 1931;24:939–53.

[64] Schöngart J, Orthmann B, Hennenberg KJ, Porembski S, Worbes M. Climate-growth relationship of tropical tree species in West Africa and their potential for climate reconstruction. Global Change Biology. 2006;12(7):1139–50.

[65] de Ridder M, van den Bulcke J, van Acker J, Beeckman H. Tree ring analysis of an African long-lived pioneer species as a tool for sustainable forest management. Forest Ecology and Management 2013;304:417–26.

[66] Souza VD, de Carvalho JOP, da Silva Mendes F, de Oliveira Melo L, Silva JNM, da Silva Jardim FC. Growth of *Manilkara huberi* and *Manilkara paraensis* after logging and silvicultural treatments in the municipality of Paragominas, Para, Brazil. 2014: Flresta, 44: 485–96.

[67] Pires JM. The Amazon forest In: Sioli H, editor. The Amazon: limnology and landscape ecology of a mighty tropical river and its basin. Dordrecht: W. Junk Publisher; 1984 p. 581–602.

[68] Rozendaal DMA, Soliz-Gamboa CC, Zuidema PA. Timber yield projections for tropical tree species: the influence of fast juvenile growth on timber volume recovery. Forest Ecology and Management. 2010;259:2292–300.

[69] Aubreville A. La foret coloniale: les foret de l'Afrique occidentale française. Ann Acad Sci Colon. 1938;9:1–245.

[70] Oldeman RAA. Tropical rain forest, architecture, silvigenesis and diversity In: Sutton SL, Whitmore TC, Chadwick AC, editors. Tropical rain forest ecology and management. Oxford: Black Scientific Oxford; 1983 p. 139–150.

[71] Worbes M, Staschel R, Roloff A, Junk WJ. Tree ring analysis reveals age structure, dynamics and wood production of a natural forest stand in Cameroon. Forest Ecology and Management. 2003;173:105–23.

[72] Baker PJ, Bunyavejchewin S, Oliver CD, Ashton PS. Disturbance history and historical stand dynamics of a seasonal tropical forest in Western Thailand. Ecological Monographs. 2005;75(3):317–43.

[73] Henry M, Besnard A, Asantee WA, Eshunf J, Adu-Bredug S, Valentini R et al Wood density, phytomass variations within and among trees, and allometric equations in a tropical rainforest of Africa. Forest Ecology and Management. 2010;260:1375–88.

[74] Santini NS, Schmitz N, Lovelock CE. Variation in wood density and anatomy in a widespread mangrove species. Trees. 2012;26:1555–63.

[75] Zanne AE, Lopez-Gonzalez G, Coomes DA, Ilic J, Jansen S., Lewis SL, Miller RB et al Global wood density database. 2009: Dryad Identifier: http://hdl.handle.net/10255/dryad.235.

[76] van Gardingen PR, Valle D, Thompson I. Evaluation of yield regulation options for primary forest in Tapajós National Forest, Brazil. Forest Ecology and Management. 2006;231:184–95.

[77] Schöngart J. The use of species-specific growth information for forest management in in central Amazonian floodplain forests In: Junk WJ, Piedade MTF, Wittmann F, Schöngart J, Parolin P (eds) Central Amazonian Floodplain Forests: Ecophysiology, Biodiversity and Sustainable Management. Dordrecht, Heidelberg, London, New York: Springer Verlag; 2010 p. 437–62.

[78] Free CM, Grogan J, Schulze MD et al (2017) Current Brazilian forest management guidelines are unsustainable for *Swietenia*, *Cedrela*, *Amburana*, and *Copaifera*: A response to da Cunha and colleagues. Forest Ecology and Management. 386:81–83. 10.1016/j.foreco.2016.09.031

