# Supplementary material for: Measures for sustainable forest management in the tropics – A tree-ring based case study on tree growth and forest dynamics in a Central Amazonian lowland moist forest
Source: PLoS One. 2019 Aug 6;14(8):e0219770. doi: 10.1371/journal.pone.0219770 (PMC6684163; doi:10.1371/journal.pone.0219770)
Supplement: S1 Table — Correlation coefficients with asterisk are significant to the p = 0.05 level. (DOCX) [file pone.0219770.s001.docx]

**Supporting Table S1**

|  | **Mean** | **STD** | **Tree height (m)** | **Age (yrs)** | **Diameter (m)** | **Entire life growth** |
| --- | --- | --- | --- | --- | --- | --- |
| **Tree height (m)** | 36.1 | 5.1 | - | - | - | - |
| **Age (yrs)** | 263 | 81 | 0.70* | - | - | - |
| **Diameter (m)** | 0.76 | 0.29 | 0.85* | 0.87* | - | - |
| **growth entire life (cm yr^-1^)** | 0.29 | 0.06 | 0.66* | 0.40 | 0.69* | - |
| **growth last 20 years (cm yr^-1^)** | 0.28 | 0.08 | 0.17 | 0.17 | 0.22 | 0.33 |
